# A Case of Pulmonary Infarction Resembling Pneumonia during Immunosuppressive Treatment for Rheumatoid Arthritis

**DOI:** 10.1155/2021/5983580

**Published:** 2021-08-18

**Authors:** Toshiki Kido, Koichiro Shinoda, Kazuyuki Tobe

**Affiliations:** First Department of Internal Medicine, University of Toyama, Toyama, Japan

## Abstract

A 67-year-old woman with rheumatoid arthritis (RA) presented with fever and dyspnea. Chest radiography and computed tomography (CT) revealed pulmonary infiltrates with ground-glass opacities. We considered bacterial or pneumocystis pneumonia because she was immunocompromised due to RA treatment. However, she had tachycardia and elevated D-dimer levels. We performed contrast-enhanced CT and subsequently diagnosed her with pulmonary embolism (PE). Though PE is not usually accompanied by parenchymal pulmonary shadows, pulmonary infarction may cause pulmonary infiltrates that can be mistaken for pneumonia. As RA is a thrombophilic disease, clinicians should be aware of PE and pneumonia as differential diagnoses in such patients.

## 1. Introduction

Pulmonary embolism (PE) is a life-threatening disease and the third leading cause of cardiovascular death after myocardial infarction and stroke [1, 2]. Pulmonary infarction is a necrosis of the lung parenchyma caused by obstruction of the pulmonary artery and occurs in a minority of patients with PE [3–5]. Although a pleural-based wedge-shaped consolidation, colloquially known as “Hampton's hump,” is a characteristic feature of pulmonary infarction on imaging, ground-glass opacity (GGO) can be an early sign of pulmonary infarction, making diagnosis difficult [6]. Here, we report a patient that presented with fever and dyspnea while receiving immunosuppressive treatment for rheumatoid arthritis (RA) with methotrexate and tacrolimus. Initially, chest imaging findings were suggestive of pneumonia; however, PE was a differential diagnosis because of the patient's clinical history. Accordingly, contrast-enhanced computed tomography (CT) was performed, and she was subsequently diagnosed with pulmonary infarction.

## 2. Case Presentation

**Figure 1:**
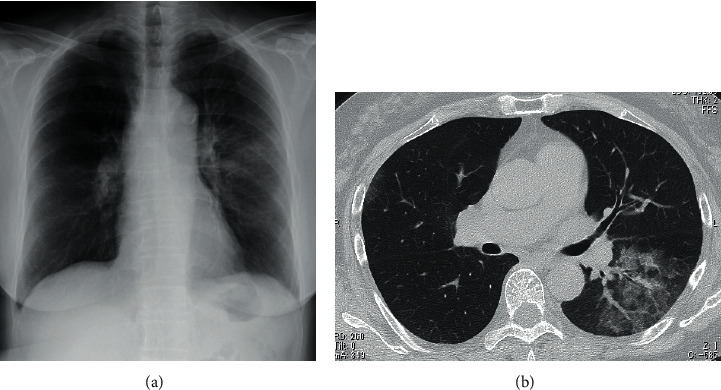
(a) Chest radiography shows subtle pulmonary infiltrate in the left lung. (b) Computed tomography (CT) reveals pulmonary infiltrates with ground-glass opacities (GGO) in the superior segment (S6) of the left lung.

**Figure 2:**
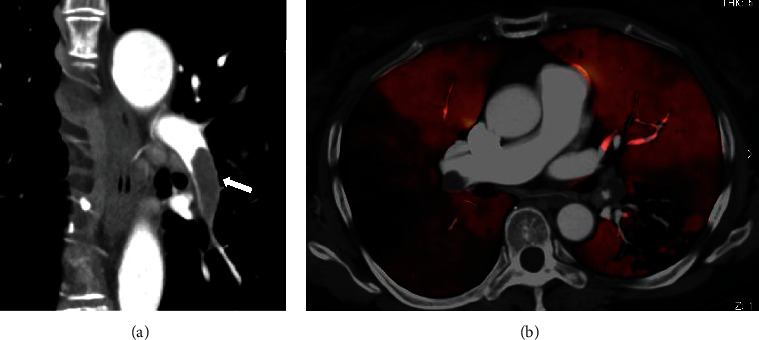
(a) Contrast-enhanced computed tomography (CT) reveals a large lesion filling the left pulmonary artery (white arrow). (b) Dual-energy CT shows areas of severe hypoperfusion in segment S4 of the right lung and segment S6 of the left lung.

A 67-year-old woman with RA visited the emergency room with a one-week history of fever and dyspnea on exertion. The patient's vitals on physical exam were as follows: blood pressure, 145/98 mmHg; heart rate, 109 beats/min; respiratory rate, 18 cycles/min; body temperature, 37.4°C; and oxygen saturation, 90%. Blood tests showed leukocytosis (9,580 cells/*μ*L) and elevated C-reactive protein (28.1 mg/L) levels. Liver and renal function tests were normal, except for elevated lactate dehydrogenase (296 IU/L). Blood gas analysis on room air showed hypoxemia (pO_2_ 51.7 Torr) and respiratory alkalosis (pH 7.495, pCO_2_ 29.6 Torr, and HCO_3_ 22.6). Electrocardiogram showed normal axis, sinus tachycardia, and a small *Q* wave in lead III without ST segment change. Radiography (Figure 1(a)) and CT (Figure 1(b)) of her chest revealed pulmonary infiltrates with GGO in the left lower lung. The patient was immunocompromised as she was receiving methotrexate (8 mg/week) and tacrolimus (1 mg/day) for the treatment of RA. We, therefore, suspected that the patient had a lower respiratory tract infection, such as bacterial or pneumocystis pneumonia. However, since the patient had tachycardia, unexpectedly severe hypoxemia given the degree of chest infiltration, and slightly elevated D-dimer levels (8.1 mg/L), a contrast-enhanced CT was performed to rule out PE. The patient was subsequently diagnosed with left deep vein thrombosis (DVT) and PE (Figure 2(a)). She was successfully treated with continuous heparin infusion and warfarin therapy. A systemic search for the cause of her thrombophilia showed no abnormalities other than mild obesity and RA. Dual-energy CT of the lesion showed a pulmonary blood flow defect in the left lung at a site consistent with the pulmonary infiltrates (Figure 2(b)). Thus, pneumonia due to her immunosuppression was not the cause of the pulmonary infiltrates. The diagnosis of pulmonary infarction was confirmed by the presence of a “Hampton's hump”-like consolidation 3 days later.

## 3. Discussion

“Hampton's hump” is a characteristic sign of PE in chest radiographs that is only seen in cases of pulmonary infarction and presents as a wedge-shaped, pleural-based consolidation [6]. In contrast, GGO-like features in the lung parenchyma, as in this case, are suggestive of two possible diagnoses. The first is mosaic perfusion, which is seen in cases of chronic pulmonary thromboembolism and is characterized by areas of GGO with hyperperfused vascular segments contrasted by areas with low attenuation due to hypoperfusion caused by vascular occlusion [6]. The second possibility is pulmonary infarction, as a fan-shaped GGO is an early sign of the condition [7]. In such cases, lung tissue ischemia leads to marked dilatation of the blood vessels in the pulmonary microcirculation, accompanied by increased vascular permeability that causes leakage of fluid and erythrocytes before tissue necrosis [6, 7]. These pathological changes likely contribute to the presence of the GGO. In this case, considering the dual-energy CT findings and the fact that her symptoms had lasted for one week, the pulmonary infiltrates with GGO likely appeared as an early sign of pulmonary infarction. The pulmonary infiltrates in our case were fan shaped, although they were not as well defined as those reported by Shinohara et al. [7]. Since the imaging findings in our case evolved to a pleural-based consolidation after 3 days, it is likely that this difference resulted from the timepoints at which the images were captured.

Treatment for RA has improved dramatically in recent years; however, attention must be paid to infections associated with immunosuppression [8]. Therefore, we initially suspected that the patient had pneumonia due to immunosuppressive therapy. Furthermore, RA is a thrombophilic disease, and there is a risk of arterial and venous thrombosis [9, 10]. D-dimer levels are slightly higher in patients with RA without clinical thrombosis than in healthy individuals [11]. Therefore, it is wrong to make a clinical decision to perform contrast-enhanced CT based on this factor alone. Rather, a negative D-dimer is useful to exclude PE in these patients [12]. The most important factor in cases of suspected PE is the physician's clinical judgement and a revised Geneva score, which is frequently used in emergency departments. Considering that the patient was >65 years old and presented with tachycardia (>95 beats/min), this patient was classified as having a moderate risk of PE according to the revised Geneva score; hence, contrast-enhanced CT was performed [13]. Although pulmonary infiltrates on chest images during RA treatment are reminiscent of respiratory tract infection or drug-induced lung injury, it should be noted that patients with RA are more prone to thromboembolic complications, such as PE and DVT, than the general population [10, 14]. Furthermore, PE leading to pulmonary infarction can also present with pulmonary infiltrates and GGO, as in this case.

## Data Availability

The datasets analyzed during the current study are available from the corresponding author on reasonable request.
